# Retraction: A phantom study to assess the reproducibility, robustness and accuracy of PET image segmentation methods against statistical fluctuations

**DOI:** 10.1371/journal.pone.0277627

**Published:** 2022-12-01

**Authors:** The PLOS ONE Editors

Following the publication of this article [1], concerns were raised regarding the authorship and ownership of the presented data. The University of Manchester investigated the authorship and data ownership concerns, and informed *PLOS ONE* that the data presented in this article were acquired at the University of Manchester by former colleagues of the author, and that the author used these data without authorization, presenting work of others as their own and excluding researchers who were involved in the data acquisition as co-authors.

In light of the institution’s findings, and in line with the institution’s recommendation, the COPE Retraction Guidelines [2], and the PLOS Plagiarism policy [3], the *PLOS ONE* Editors retract this article.

Since the author did not have authorization of the data owner(s) to publish the data and study information reported in this article, the retracted *PLOS ONE* article was removed from the *PLOS ONE* website at the time of retraction. The article’s Copyright and Data Availability statements were updated at the time of retraction and removal, and the removed contents are no longer offered under the Creative Commons Attribution License.

The author did not agree with the retraction.
